# Supplementary material for: Algorithm for using dual energy computed tomography to determine chemical composition: A feasibility study
Source: PLoS One. 2025 Jun 30;20(6):e0322805. doi: 10.1371/journal.pone.0322805 (PMC12208427; doi:10.1371/journal.pone.0322805)
Supplement: S1 File — Process for obtaining representative energy from 140 kVp spectral energy for H₂O using SpekCalc and Geant4 simulation. (DOCX) [file pone.0322805.s001.docx]

**Supplement document**

<A process for obtaining representative energy from 140 kVp spectral energy for H_2_O>

Step 1. Acquisition of energy spectrum using SpekCalc. Energy spectral values of 15.2–139.9 kVp were obtained with an interval of 0.1 kVp energy.

Step 2. Apply the acquired energy spectrum (acquired by step 1) to perform spectral energy simulation using Geant4 and acquire MAC. MAC is obtained through the formula $I=I_{0}e^{-\mu t}$ where $I_{0}$ is the number of simulation events, $\mu$ is attenuation coefficient, $t$ is the thickness of the material, and $I$ is the number of events counted by the detector. The resulting MAC for H_2_O is 0.2274 for simulations using the spectral energy of 140 kVp.

Step 3. Identify the representative energy corresponding to the MAC obtained in Step 2 by interpolating the relationship between energy and MAC of NIST data using MATLAB. The representative energy for 140 kVp obtained by the abovementioned method is 49.8 keV.

Step 4. Perform representative energy simulation by applying the representative energy and obtain MAC. The resultant MAC for H_2_O is 0.2269 for simulations using the mono energy of 49.8 keV.

Step 5. Comparing the mass attenuation coefficient obtained in Steps 2 and 4 and determining whether the mono energy reported is appropriate to represent the energy spectrum. The percentage difference of MACs obtained because of Genat4 simulation of spectral energy and mono energy is 0.05%, which is sufficiently reliable.
