# Supplementary material for: Algorithm for using dual energy computed tomography to determine chemical composition: A feasibility study
Source: PLoS One. 2025 Jun 30;20(6):e0322805. doi: 10.1371/journal.pone.0322805 (PMC12208427; doi:10.1371/journal.pone.0322805)
Supplement: Attachment — Submitted filename: Reviewer comment_final.pdf [file pone.0322805.s005.pdf]

PLOS ONE

Manuscript Number: PONE-D-24-42552

Full Title: Algorithm for Using Dual Energy Computed Tomography to Determine Chemical Composition: A Feasibility Study

General comment to the author's:

The study is well-prepared and has significant results on an important topic. But some revisions are required. The specific comments are also given in the .doc file of the manuscript as comments. It is also necessary to correct some technical mistakes in the manuscript (reference citation and full stop before reference citation).

Specific comment:

1. **Lines 35-36: "For mono-energetic X-rays, the differences were < 0.01% for compounds and 6.02% for mixture, respectively" I think no need «respectively» at the end of the sentence.**

The manuscript has been revised according to your suggestion.

L35: For mono-energetic X-rays, the differences were < 0.01% for compounds and 6.02% for mixture.

2. **Line 111: Precisely, which material the authors are used to fill this Box shaped material in GEANT4 simulation?**

Thank you for your comment. In this study, box-shaped materials were individually modeled for elements with atomic numbers ranging from 1 to 20 in the Geant4 simulation. This approach was employed to evaluate the X-ray attenuation characteristics of different elements. We will clarify this information in the manuscript to ensure better understanding.

Sentence to be added in the manuscript:
L113: In the Geant4 simulation, each box-shaped material was filled with a single element corresponding to atomic numbers ranging from 1 to 20 to obtain MACs data for individual elements.

3. **Lines 125-126: the author hereby stated that: "In principle, applying the algorithm for identifying chemical constituent from spectral energy, we should integrate over the complete energy spectrum".How the energy spectrum was obtained?**

Thank you for your insightful question. In this statement, we were referring to the theoretical approach of integrating over the complete energy spectrum to identify chemical constituents.

To validate the selection of representative energy, we compared the MACs obtained from the full energy spectrum with those derived from specific monoenergies representing the spectral X-ray. The representative energy was determined based on the condition that the MAC of a specific reference material (H_2O) matched the MAC integrated over the full energy spectrum.

This method was further validated using PMMA and cortical bone (ICRP) to ensure consistency across different tissue-equivalent materials.

For further details on how the representative energy for H_2O, PMMA, and cortical bone was determined, please refer to the later sections of the manuscript and supplement document.

Added Sentence

L139: To provide a detailed explanation of the process for obtaining representative energy, a supplementary document has been included. This document outlines the step-by-step methodology used to determine the representative energy for 140 kVp spectral energy, specifically for H_2O, using SpekCalc, Geant4 simulations, and NIST data interpolation.

4. **Line 127: I suggest to change the word "Monoenergy" in the title to "monoenergy".**
We have revised the word by changing "Monoenergy" to "monoenergy" as suggested.

5. **Lines 165-166: "However, the noise in the image and uncertainty in measurement, $f(z)$ would not vanish but become close to 0" What are the underlying factors contributing to noise and uncertainty in image data?**

Thank you for your comment. Considering the possibility that the explanation of noise and measurement uncertainty might not be sufficiently clear, we have added a sentence indicating that this topic is discussed in detail in the Discussion section. This revision ensures that readers can better understand the discussion regarding noise and measurement uncertainty.

Added sentence:

L174: The factors contributing to noise in the image and measurement uncertainty are discussed in the Discussion section.

The main factors contributing to noise and uncertainty in image data in this study are as follows:

1. **Noise and uncertainty in CT scanning**
   - **Evaluation of noise impact:** In this study, we analyzed the effect of CT image noise on the performance of our algorithm. Hünemohr et al. evaluated weight fraction estimation by incorporating Gaussian-distributed noise into electron density and the effective atomic number (Zeff), demonstrating prediction errors across different elements **(Discussion, Evaluating the effect of noise section)**.
   - **Mitigating noise effects using PCA:** Lalonde and Bouchard proposed using a larger number of energy spectra than principal components (PC) in principal component analysis (PCA) to mitigate the effect of noise. In our study, we also assessed whether the developed algorithm could accurately determine elemental composition in noisy conditions **(Discussion, Evaluating the effect of noise section)**.

2. **Uncertainty in mass attenuation coefficients (MACs)**

    - The MAC is derived from HU values obtained via CT scans, and noise and measurement errors can be introduced in this process. According to the International Atomic Energy Agency (IAEA), CT values should remain within ±20 HU of the manufacturer's recommended values, leading to approximately 2% uncertainty in MACs relative to water as the reference **(Discussion, Evaluating the effect of noise section)**.

    - To ensure a conservative assessment, we set an upper error limit of 3% in our experiments. If an error exceeding 3% is introduced into the MAC, the fractional weight calculated by our algorithm exhibited an additional error exceeding 5% **(Discussion, Evaluating the effect of noise section)**.

3. **Statistical error in Geant4 simulations**

    - The built-in and input data for the algorithm were obtained through Geant4 simulations. To assess statistical reliability, we varied the number of events and calculated MAC values multiple times. The results showed that with 1E+08 events, the standard deviation was 5.68E-05, and the relative error remained below 0.01, confirming the statistical reliability of the simulation **(Discussion, Statistical error of Geant4 simulation section)**.

These factors contribute to noise and uncertainty in image data, and our study has taken them into account to evaluate the reliability of the proposed algorithm.

6. Line 195: I suggest to change the word "Utilized" to "used"

We have revised the text by changing **"Utilized" to "used"** as suggested.

7. Lines 208-209: The authors mentioned "the formulas mentioned in 2.2.1 and 2.2.2" but there are not these formulas in all the manuscript.

Thank you for your comment. We have identified that referring to "the formulas mentioned in 2.2.1 and 2.2.2" could be misleading since these specific section numbers do not exist in the manuscript. To enhance clarity, we have revised the phrase to **"the formulas mentioned in the Theory section eq 1 to 6."** This ensures that readers can properly refer to the relevant theoretical background.

L219: As an example of identifying the atomic number of an unknown material through the formulas mentioned in the Theory section eq 1 to 6.

8. Lines 275-277: "It was confirmed that the %difference in the case of number of constituents was 4, 5 and 6 were 0.340 – 5.449%, 1.410 – 5.188%, and 3.693 – 6.027%, respectively" What does the author intend to convey? If possible, rephrase this sentence for more clarity.

Thank you for your comment. We have revised the sentence to clarify the range of %difference for different numbers of constituents in the mixtures. This modification ensures that readers can better understand the presented data and its implications.

Revised Sentence

L296: The results in Table 3 show that the %difference ranged from 0.340% to 5.449% for mixtures with four constituents, 1.410% to 5.188% for five constituents, and 3.693% to 6.027% for six constituents. This indicates that as the number of constituents increases, the overall %difference tends to increase. The reasons for this will be discussed in the Discussion section.

9. **Lines 307-308: "it is considered a useful result, but several considerations were made about the problems encountered during performing of this study and are described below." I suggest to rephrase this sentence to enhance the clarity and convey the intended meaning more precisely.**

Thank you for your comment. We have revised the sentence to improve clarity and ensure that the intended meaning is conveyed more precisely.

Revised Sentence

L323: The originality of our algorithm lies in its ability to determine the constituent elements and their fractional weights for an unknown material containing two or more elements, using only two CT energy spectra. Unlike conventional approaches that approximate material composition using Zeff, our method directly estimates elemental fractions. However, challenges remain in accurately interpreting CT images due to inherent noise and measurement uncertainties. In particular, image noise and variations in CT number can affect the reliability of elemental composition analysis.

10. **Line 313: I suggest to change the word "quantized" to "quantified"**

Thank you for your suggestion. We have revised the text by changing "quantized" to "quantified" as recommended.

11. **Line 232: in the phrase «The algorithm was tested on five compounds that combine C, H, O elements...» which compounds? I suggest to cite them.**

Thank you for your valuable comment. We have revised the text to specify the five compounds used in our evaluation: H_2O (water), C_2H_4 (ethylene), C_8H_8 (styrene), $C_5O_2H_8$ (valeric acid), and $C_{10}H_8O_4$ (naphthalic acid).

Revised Sentence

L329: The algorithm was tested on five compounds—H_2O (water), C_2H_4 (ethylene), C_8H_8 (styrene), $C_5O_2H_8$ (valeric acid), and $C_{10}H_8O_4$ (naphthalic acid)—which contain C, H, and O elements, as well as nine mixtures of the body tissue materials mentioned [18, 19].

12. **Lines 233-234: "In the case of body tissue materials, the fractional weight of some elements was slightly modified" which elements? I suggest to enumerate the elements**

Thank you for your valuable comment. We have revised the text to specify the elements whose fractional weights were modified. In the case of body tissue materials, the fractional weight of hydrogen (H) and carbon (C) was slightly adjusted to ensure that the sum of all fractional weights equaled 100%.

Revised Sentence

L241: In the case of body tissue materials, the fractional weight of hydrogen (H) and carbon (C) was slightly modified to ensure that the total fractional weight summed to 100%.

13. **Line 390-391: The authors states that to acquire both data, 1E+08 events were used for the simulation. To confirm whether data is statistically reliable, the number of events was changed and MACs was obtained ten times for each event" Why the events number was changed? There are some methods that explain this?**

Thank you for your question. The number of events was varied to evaluate the statistical reliability of the Monte Carlo simulation results. In Monte Carlo-based simulations, statistical fluctuations are inherent, and increasing the number of events generally reduces these fluctuations, improving the precision of estimated parameters such as the Mass Attenuation Coefficients (MACs). To ensure that our results were not significantly affected by statistical noise, we systematically changed the number of events and analyzed how it impacted the computed MAC values. This approach aligns with standard practices in Monte Carlo simulations for assessing convergence and stability.

14. **If not, how did the authors go about changing the number of events, given that they defined 108 to obtain the data?**

The number of events was modified incrementally by setting different predefined values (1E+03, 1E+04, 1E+05, 1E+06, 1E+07, 5E+07, and 1E+08) and running the simulation separately for each case. This allowed us to observe the impact of event count on MAC stability. The results from each event configuration were compared to determine the minimum number of events required to achieve statistical reliability. This method follows common Monte Carlo validation techniques used in radiation transport simulations.

15. **For each event, MACs were obtained ten times. For each defined energy, there must be a MAC value. If the MAC value for an event is obtained ten times then total MAC is the average (for an energy or intensity)?**

Yes, for each event configuration, MAC values were obtained ten times, and for each defined energy level, an individual MAC value was computed. The final MAC value for a given

energy was calculated as the average of these ten values. This averaging process reduces statistical fluctuations and provides a more stable estimate of the MAC for each energy level. The standard deviation and relative error of these ten values were also analyzed to quantify the uncertainty and ensure the robustness of our results.

16. Line 457: I suggest that the authors start this by "find the space …"

Thank you for your suggestion. We have revised the sentence to begin with "Find the space," as recommended. This modification enhances clarity and aligns with the intended meaning of the section.

Revised Sentence

L483: Firstly, find the space of relevant elements by refining the search space to focus on a subset of elements more likely to be present in the unknown material ~

Editor comments:

In this manuscript, Choi et al. report on the use of dual-energy computed tomography (CT) to develop an algorithm to identify the chemical constituents of an unknown material (compound or mixture) and improve the accuracy of material discrimination. Using dual energies 80/140 kVp for spectral CT scans in addition to a set of 50/80 and 80/100 keV for mono-energetic X-rays, their algorithm correctly determined the chemical constituent elements of unknown materials. Their funding was compared to the data provided by the National Institute of Standards and Technology (NIST) and displayed differences of $< 0.01\%$ for compounds and 6.02% for mixture in the case of mono-energetic X-rays, and 2.98% for compounds and 6.03% for mixtures for spectral X-rays, respectively

The research has yielded important insights that make it essential for the manuscript to be published in PlosOne. The following reasons support this recommendation:

- Firstly, the research is both original and relevant. It offers valuable information on an important step towards the development of an algorithm for the evaluation of the stopping power ratio (SPR), which is crucial for the calculation of the physical dose of particle radiation in the treatment planning system (TPS). The findings are of great importance to researchers and medical staff involved in CT information in the volume unit. The study can be of great interest if the clinical applicability is demonstrated and concrete outcomes are presented.
- Secondly, the research was conducted with a high level of scientific rigour. Both qualitative and quantitative methods were used to collect and analyze data, enhancing the reliability and validity of the findings. Geant4 results were compared to the existing NIST data base.

General comments:

1. Upon assessing the similarity level of your manuscript, it has been observed that approximately 60% of the content of the main text is derived from your paper posted in the Researchsquare repository (https://doi.org/10.21203/rs.3.rs-2593701/v1). While it is permissible to submit the manuscript to an open archive while it is under review, it is advisable to have the same manuscript submitted to PlosOne in the repository, which would result in an approximate 100% similarity with the file in the repository. This

adherence to scientific publication ethics and policy aims to prevent dual submission. Should you have any reasons to disclose to the editorial board of PlosOne regarding this matter, please do so.

We would like to clarify that this manuscript has not been submitted to any other journal apart from PLOS ONE, and there is no case of dual submission.

The paper posted in the ResearchSquare repository is a preprint version, which was uploaded to share our research findings with the academic community and facilitate discussions. This aligns with PLOS ONE's preprint policy and follows the appropriate submission process.

The preprint version remains unpublished and serves solely as an early-stage academic disclosure.

Please let us know if any further clarification is required.

2. The title of the manuscript needs to be revised considering changing the expression "a feasibility study"

Thank you for your comment. Based on your suggestion, we have revised the manuscript title by replacing "a feasibility study" with "Method Development and Validation" to provide a clearer and more precise description of the study.

This modification highlights that our research is not merely a feasibility assessment but also includes algorithm development and validation.

Revised title

L2: Algorithm for Using Dual-Energy Computed Tomography to Determine Chemical Composition: Method Development and Validation

3. The manuscript's sections need to be renumbered, as there is no numbered section whereas the authors keep referring to sections. E.g. line 209 "mentioned in 2.2.1 and 2.2.2"

Thank you for your comment. Upon reviewing the manuscript, we identified inconsistencies in section numbering and have revised these references accordingly.

Instead of using section numbers, we have replaced them with the corresponding section to ensure clarity and ease of navigation for readers.

This modification allows for a more consistent and user-friendly reading experience.

Revised Sentence

L219: As an example of identifying the atomic number of an unknown material through the formulas mentioned in the Theory section eq 1 to 6.

4. In addition, some grammatical and sentence structure issues should be addressed during the review process as well as subscript and superscript notations. (e.g. Line 318: "… for the* detection of COVID-19 …"; Line 324: "… regarded as* excellent."; Line 325: "… the full doses*"

Thank you for your comment. We have thoroughly reviewed the manuscript and corrected any errors in subscript and superscript notations. Additionally, we have identified and revised grammatical errors and sentence structure issues to improve the clarity and readability of the manuscript.

Specific comments:

1. **Title:** Need to be revised to convey the main purpose of the study

   Thank you for your comment. Based on your suggestion, we have revised the manuscript title by replacing "a feasibility study" with "Method Development and Validation" to provide a clearer and more precise description of the study.

   This modification highlights that our research is not merely a feasibility assessment but also includes algorithm development and validation.

   Revised title

   L2: Algorithm for Using Dual-Energy Computed Tomography to Determine Chemical Composition: Method Development and Validation

2. **Abstract:** This section is for a large readership and should be written appropriately, avoiding abbreviations not acknowledged by the general public as much as possible (SPR). Consider clearly explaining where its use is unavoidable or define it for the first occurrence, and use it thereafter.

   Thank you for your valuable comment. We have revised the abstract to ensure that abbreviations unfamiliar to a general readership are either explained at their first occurrence or used in a way that maintains clarity. Specifically, "SPR" is now introduced as "stopping-power ratio (SPR)" before being used subsequently in the text. We appreciate your feedback in improving the readability of our abstract.

   Revised Abstract

   L25: Using dual-energy computed tomography (CT), this study aims to develop an algorithm to identify the chemical constituents of an unknown material (compound or mixture) and improve the accuracy of material discrimination. The algorithm requires mass attenuation coefficients (MAC), which were obtained using a dual-energy CT as input, to identify the elemental composition and calculate its weight fraction. To evaluate the functionality of the developed algorithm, it was applied to determine the chemical constituents of human tissues. Furthermore, the results were compared with those provided by the National Institute of Standards and Technology (NIST). We used dual energies of 80/140 kVp for spectral CT scans as inputs to the algorithm, in addition to a set of 50/80 and 80/100 keV for mono-energetic X-rays. The algorithm correctly determined the chemical constituent elements of unknown materials. Results were obtained for the fractional weights of each component for mono-energetic X-rays and spectral X-ray use. For mono-energetic X-rays, the differences were < 0.01% for compounds and 6.02% for mixtures. For spectral X-rays, the differences were 2.98% for compounds and 6.03% for mixtures. We developed an algorithm to determine the type and weight fraction of an element using the MAC of dual-energy CT. The algorithm can exclude the inherent uncertainty in stopping-power ratio (SPR) calculations, which are crucial for proton therapy, and improve the accuracy of dose calculations in radiation therapy planning.

3. **Materials and methods:**

① Line 103: what do the authors mean by "…when a CT image comprising multiple voxels is applied to the algorithm"

Thank you for your insightful comment. After careful discussion, we have determined that the phrase "…when a CT image comprising multiple voxels is applied to the algorithm" may cause confusion for readers. As it does not affect the overall flow or clarity of the manuscript, we have decided to remove this phrase from the text.

We appreciate your feedback, which has helped improve the readability and precision of our manuscript.

② Line 110: "Cylindrical gamma detector: radius of 0.1 mm × height of 0.1 mm ". Is the description of the detector used in this research realistic and implemented in real experiments?

Thank you for your insightful question. The cylindrical gamma detector described in our study, with a radius of 0.1 mm and a height of 0.1 mm, was chosen to ensure high spatial resolution in the Monte Carlo (MC) simulation. The dimensions were deliberately set to minimize the volume of interaction and accurately capture the attenuation characteristics of photons passing through the material.

While such a detector configuration may not be commonly implemented in conventional experimental setups, it is a standard practice in Geant4-based MC simulations to use small-scale virtual detectors to improve statistical accuracy and reduce potential spatial averaging effects. The purpose of this setup is to obtain precise mass attenuation coefficients (MACs) without artifacts from detector geometry, ensuring that the data can be reliably compared with reference values from the National Institute of Standards and Technology (NIST).

In practical experimental implementations, detectors with larger active areas are typically used, and their spatial resolution is compensated through appropriate calibration procedures. However, in the simulation, the use of a small-scale detector does not compromise the accuracy of the MAC computation, as it solely serves to quantify photon interactions at a localized level.

Thus, while the detector dimensions in the simulation may differ from those in real-world experimental setups, this choice is methodologically justified for the purpose of achieving high-precision MAC calculations in a controlled virtual environment.

③ Lines 114-115: "In this study, CT images based both on full energy spectra simulations and mono-energic beams were simulated. " How did you simulate CT images?

Thank you for your insightful comment. We acknowledge the ambiguity in our original statement and have revised it for clarity. In this study, we did not directly simulate CT images. Instead, we performed Monte Carlo (MC) simulations to obtain mass attenuation coefficients (MACs) for materials with atomic numbers ranging from 1 to 20. These simulations were conducted using both full energy spectra and mono-energetic beams.

Revised Sentence

L117: In this study, we conducted Monte Carlo (MC) simulations to obtain mass attenuation coefficients (MACs) for materials with atomic numbers ranging from 1 to 20, using both full energy spectra and mono-energetic beams.

④ Figure 1 is confusing. The caption should be revised. The track in Fig. 1 (b) shows that the statistic was too low and the derived data's uncertainties are expected to be top high. Is the position of the source in (a) the same as in (b)? In (b), the source seems to be emitting from inside the box whereas in (a), it is located at a distance of 10.5 cm.

Thank you for your valuable comment. We acknowledge the potential confusion regarding Figure 1 and have revised the caption to provide better clarity.

In Figure 1(a), the schematic illustrates the final X-ray paths reaching the detector based on the initial source position. The arrangement in (b) is not identical to (a); rather, it serves as a visualization of X-ray tracks in the simulation. To ensure the visibility of individual photon tracks, we intentionally reduced the number of simulated photons in (b). If the full 1E+08 photons used in the actual simulation were displayed, the entire screen would be filled with beams, making it impossible to distinguish individual photon steps. The revised caption clarifies these aspects for better understanding.

Revised Caption

L123: Fig 1. (a) Geant4 modeling for calculating mass attenuation coefficients. The blue box represents the object used to obtain mass attenuation coefficients and has a thickness of 1 cm. (b) Visualization of X-ray tracks in a simulation. To enhance visibility, fewer photons were displayed; using 1E+08 photons, as in the actual simulation, would obscure individual photon tracks. The source position in (b) differs from (a) but follows the same beam path concept.

⑤ Lines 142 - 154: the detailed description could be simplified or added as supplementary material as it does not contribute to the research's main objective.

Thank you for your valuable feedback. We understand your suggestion to simplify the detailed description or move it to supplementary material. However, as this study focuses on the development of an algorithm, mathematical formulation, including the derivation of the objective function, is a fundamental component of the manuscript. These equations

are essential for explaining how the algorithm identifies the atomic number and weight fractions of unknown materials.

Removing or significantly simplifying this section may make it difficult for readers to fully understand the underlying methodology and the theoretical framework supporting the algorithm. Given its importance to the core contribution of our research, we respectfully request to keep this section within the main text.

⑥ Fig. 2. The last step "<5% difference with input attenuations ◊ output: Final solutions" is confusing. It might lead to an infinite loop if the target of <5% is never reached. Another issue is that you have data with 6%, which questions whether the authors used this algorithm for the data presented in the paper.

Thank you for your insightful comment. The concern regarding a potential infinite loop is valid; however, in our algorithm, the threshold of <5% difference is not a strict stopping criterion. When the number of constituent elements increases, the overall uncertainty tends to increase as well. To account for this, the algorithm allows for an adaptive threshold extension beyond 5% when necessary.

Specifically, if the difference exceeds 5% but remains within a reasonable range, the algorithm continues evaluating potential solutions instead of terminating indefinitely. This approach ensures that materials with complex compositions can still be analyzed effectively without being constrained by an overly rigid threshold. In cases where the difference remains too high, the algorithm excludes those solutions from the final output.

Regarding the presence of data with a 6% difference, this value appears in the results because the algorithm also outputs all cases where the uncertainty exceeds 5%. Although such cases are marked as failures in the algorithm's internal logic, they are still included in the output for completeness and transparency. The threshold extension does not compromise the accuracy of the results but rather improves the robustness of the material identification process.

We have added this sentence to the manuscript to clarify the handling of cases where uncertainty exceeds 5%.

Added Caption

L208: The algorithm is designed to terminate when the difference in input attenuation coefficients converges within 5%. However, for cases where the uncertainty exceeds 5%, the algorithm allows for the optional collection of failed output.

⑦ Line 168: Make sure all captions appear in the manuscript in the order of their citation in the text. Fig.4 seems to be quoted whereas Fig. 3 is not yet mentioned.

Thank you for your suggestion. We have revised the sentence by removing the reference to Fig. 4 to maintain the proper order of figure citations in the manuscript. The updated

sentence now reads. This modification ensures that the figures appear in the correct sequence, preventing any potential confusion for the readers.

Revised Sentence

L176: For robustness against noise, we consider multiple local extrema during the process of minimization.

⑧ Line 209: “mentioned in 2.2.1 and 2.2.2” ?

Thank you for your comment. Upon reviewing the manuscript, we identified inconsistencies in section numbering and have revised these references accordingly.

Instead of using section numbers, we have replaced them with the corresponding section to ensure clarity and ease of navigation for readers. This modification allows for a more consistent and user-friendly reading experience.

Revised Sentence

L219: As an example of identifying the atomic number of an unknown material through the formulas mentioned in the Theory section.

⑨ Lines 212-215: This description should be enhanced taking into account the update in Fig. 4 caption.

Thank you for your valuable comment. We have updated the caption of Fig. 4 for better clarity and alignment with the manuscript description.

Revised Sentence

L232: Fig 4. Identification process of polymethyl methacrylate ($C_5O_2H_8$) using the algorithm. The graph shows the objective function and fractional weight curves during the identification of the third chemical constituent, following the assumption of hydrogen (H) as the first element (step 2 in Fig. 2). The trial element, carbon (C, Z = 6), led the objective function to a local minimum at Z = 8, which was selected as the third element. Other singularities were rejected based on the constraint that fractional weights must remain within the range [0,1].

⑩ Lines 228-230: “In the case of body tissue materials, the fractional weight of some elements was slightly modified so that the sum of the fractional weights was 100%.” Why did the authors modify the fractional weight of some elements? What was the rationale behind the modification? This is crucial to the end users and readers of the paper.

Thank you for your insightful question. The modification of fractional weights for certain elements occurs as part of the final tuning step in the algorithm. For instance, if hydrogen

is determined to be 20% and oxygen 79.8%, a 0.2% discrepancy arises. This tuning process is necessary to ensure that the total fractional weight sums to exactly 100%.

This adjustment is fundamentally a result of the uncertainty inherent in the input mass attenuation coefficients (MACs). Since MAC values carry a degree of uncertainty, the algorithm incorporates a fine-tuning process that slightly adjusts these values to minimize final errors in material composition estimation. By iteratively refining the input MACs within a reasonable range, the algorithm ensures that the computed fractional weights remain as accurate as possible.

Revised Sentence

L241: In the case of body tissue materials, the fractional weight of hydrogen (H) and carbon (C) was slightly modified to ensure that the total fractional weight summed to 100%. This adjustment was necessary because small discrepancies can arise due to rounding errors or variations in reported elemental compositions in reference databases. Additionally, a fine-tuning process is applied in the final stage of the algorithm to correct minor discrepancies. For instance, if hydrogen is determined to be 20% and oxygen 79.8%, a 0.2% discrepancy arises. This tuning process ensures that the total fractional weight sums precisely to 100%. This adjustment is primarily a result of the inherent uncertainty in the input mass attenuation coefficients (MACs). Since MAC values contain small uncertainties, the algorithm incorporates an iterative fine-tuning process that slightly adjusts these values to minimize final errors in material composition estimation. By refining the input MACs within a reasonable range, the algorithm ensures that the computed fractional weights remain as accurate as possible while preserving the original material composition.

4. **Results:**
Table 2 caption should be revised to consider the content of the table itself.

Thank you for your valuable comment. We have revised the caption of Table 2 to better reflect the content of the table. The updated caption provides a clearer summary of the results, emphasizing the evaluation of percentage differences in fractional weights for various compounds and the accuracy of the algorithm in identifying chemical constituents. We appreciate your feedback in improving the clarity of our manuscript.

Revised Caption

L289: Table 2. Comparison of chemical constituent identification results using the algorithm with dual-energy mass attenuation coefficients. The percentage differences in fractional weights were evaluated for compounds containing different numbers of elements, demonstrating the accuracy of the algorithm in identifying chemical compositions.

Line 274: “The reasons for this will be discussed in more detail in section 4.1.”?

Thank you for pointing this out. We apologize for any confusion caused and appreciate your feedback in improving the clarity of our manuscript.

Revised Sentence

L299: The reasons for this will be discussed in the Discussion section.

Lines 334-336: What is the contribution to the scientific research presented?

Thank you for your insightful comment. The statement regarding computation time is crucial to the scientific contribution of our research, as it highlights the algorithm's feasibility for practical applications, particularly in medical imaging. While the algorithm successfully determines material composition, its computational efficiency remains a key consideration for large-scale implementation, such as processing medical images with hundreds of slices.

By reporting the current computation time, we establish a baseline for future optimizations in both hardware and software. This information underscores the need for improvements in processing speed to ensure real-time applicability in clinical settings. Addressing computational limitations is a critical step toward translating our methodology into practical use, making this discussion an important part of our research findings.

Lines 338-339: "Alternatively, we can reduce the number of voxels by skipping the calculation of nearby and similar HU-valued voxels." What is the direct consequence on the obtained results, if implemented?

Thank you for your valuable question. If the proposed voxel reduction approach is implemented, the direct consequence would be a significant improvement in computational efficiency. By skipping calculations for neighboring voxels with similar HU values, the total number of required computations can be greatly reduced. This is based on the assumption that adjacent voxels with similar HU values are highly likely to belong to the same material, making redundant calculations unnecessary.

Revised Sentence

L363: This approach is expected to significantly reduce computation time while maintaining overall accuracy, as neighboring voxels with similar HU values are likely to have the same material composition.

Line 407: Is "three to six components" a limitation of the study? What is the rationale for setting this limit?

Thank you for your insightful question. The range of "three to six components" is not an inherent limitation of the algorithm but rather a practical choice based on the scope of this study. The rationale for this range is threefold:

1. Computational Feasibility and Complexity Management

Three to six components represent a reasonable complexity level for evaluating the algorithm's ability to identify chemical compositions while ensuring computational feasibility. As the number of components increases, the complexity of solving for fractional weights also increases exponentially. Therefore, this range allows us to validate the accuracy and reliability of the algorithm without excessive computational burden.

2. Reference Literature and Known Elemental Compositions

Upon reviewing reference literature that provides known elemental compositions and their fractional weights, we found that materials with more than six major components are rare. Most reported materials relevant to our study contain six or fewer primary elements, making this range both practical and representative for evaluation.

3. Significance of Minor Elemental Contributions

In certain materials, specific elements are present only in trace amounts. These minor constituents do not significantly influence the overall material composition and are not ideal for evaluating the algorithm's performance. By focusing on materials with three to six dominant components, we ensure that the algorithm is tested on cases where each constituent plays a meaningful role in the composition.

We appreciate your comment and have updated the manuscript to clarify this point.

5. **Conclusions:**
Please, summarize the main findings of your research.

Thank you for your valuable suggestion. We have revised the conclusion to provide a clearer summary of our main research findings.

Revised Sentence

L504: We developed an algorithm that determines element types and their weight fractions using the MACs of dual-energy X-rays. The algorithm accurately identified elemental compositions and fractional weights for materials with up to six constituents, demonstrating its effectiveness in material characterization. While some uncertainty remains, our findings emphasize the need for further refinement to enhance accuracy and robustness.
