# Supplementary material for: Algorithm for using dual energy computed tomography to determine chemical composition: A feasibility study
Source: PLoS One. 2025 Jun 30;20(6):e0322805. doi: 10.1371/journal.pone.0322805 (PMC12208427; doi:10.1371/journal.pone.0322805)
Supplement: Attachment — Submitted filename: Reviewer comment.docx [file pone.0322805.s003.docx]

**PLOS ONE**

**Manuscript Number: PONE-D-24-42552**

**Full Title: Algorithm for Using Dual Energy Computed Tomography to Determine Chemical Composition: A Feasibility Study**

**General comment to the author’s:**

The study is well-prepared and has significant results on an important topic. But some revisions are required. The specific comments are also given in the .doc file of the manuscript as comments. It is also necessary to correct some technical mistakes in the manuscript (reference citation and full stop before reference citation).

Specific comment:

Lines 35-36: “For mono-energetic X-rays, the differences were < 0.01% for compounds and 6.02% for mixture, respectively” I think no need «respectively» at the end of the sentence.

Line 111: Precisely, which material the authors are used to fill this Box shaped material in GEANT4 simulation?

Lines 125-126: the author hereby stated that: “In principle, applying the algorithm for identifying chemical constituent from spectral energy, we should integrate over the complete energy spectrum”.How the energy spectrum was obtained?

Line 127: I suggest to change the word "Mono energy" in the title to "monoenergy”.

Lines 165-166: “However, the noise in the image and uncertainty in measurement, $f(z)$ would not vanish but become close to 0” What are the underlying factors contributing to noise and uncertainty in image data?

Line 195: I suggest to change the word “Utilized” to “used”

Lines 208-209: The authors mentioned “the formulas mentioned in 2.2.1 and 2.2.2” but there are not these formulas in all the manuscript.

I suggest to change the word "mono energies" to "monoenergies” in all the text

Lines 275-277: “It was confirmed that the %difference in the case of number of constituents was 4, 5 and 6 were 0.340 – 5.449%, 1.410 – 5.188%, and 3.693 – 6.027%, respectively” What does the author intend to convey? If possible, rephrase this sentence for more clarity.

Lines 307-308: “it is considered a useful result, but several considerations were made about the problems encountered during performing of this study and are described below.” I suggest to rephrase this sentence to enhance the clarity and convey the intended meaning more precisely.

Line 313: I suggest to change the word “quantized” to “quantified”

Line 232: in the phrase «The algorithm was tested on five compounds that combine C, H, O elements...» which compounds? I suggest to cite them.

Lines 233-234: “In the case of body tissue materials, the fractional weight of some elements was slightly modified” which elements? I suggest to enumerate the elements

Line 390-391: The authors states that to acquire both data, 1E+08 events were used for the simulation. To confirm whether data is statistically reliable, the number of events was changed and MACs was obtained ten times for each event” Why the events number was changed? There are some methods that explain this?

If not, how did the authors go about changing the number of events, given that they defined 108 to obtain the data?

For each event, MACs were obtained ten times. For each defined energy, there must be a MAC value. If the MAC value for an event is obtained ten times then total MAC is the average (for an energy or intensity)?

Line 457: I suggest that the authors start this by “find the space …”
